# The insurability of innovative pharmaceutical cancer technologies

**DOI:** 10.1186/s13584-020-00426-w

**Published:** 2020-12-21

**Authors:** Shuli Brammli Greenberg, Einat Dotan, Rachel Arazi

**Affiliations:** 1 The Department of Management and Health Economics, School of Public Health, Hebrew University of Jerusalem, Jerusalem, Israel; 2 Myers-JDC Brookdale institute, Jerusalem, Israel; 3 School of Public Health, University of Haifa, Haifa, Israel; 4 The Department of Business Management, Ono Academic College, Kiryat Ono, Israel

**Keywords:** Insurability, Uncertainty, Pharmaceutical Cancer technologies, Drug Price

## Abstract

The scientific literature, including several papers published in the IJHPR, has raised the issue of the spiraling cost of cancer treatment, including that of cancer drugs and other technologies such as gene and cell therapies. In this perspective, we review three criteria for insurability and show that they may not be met.

First, the uncertain trends in the cost of innovative pharmaceutical and other cancer technologies make the *maximum possible loss per event* very difficult to predict and to manage in terms of insurer solvency. Second, the uncertainty of the price, the period that a drug or other cancer care technology will be administered and the number of individuals that will need the technology makes it difficult to predict future *insurance premiums* and whether they will be affordable to the target population*.* Third, *public coverage needs to be consistent with societal values*. However, pressure to limit public coverage will gradually increase as the possibilities of innovative pharmaceutical cancer technologies expand, thus transferring the burden onto commercial insurance. This is a phenomenon that is virtually impossible to predict accurately, but which will certainly undermine the status of health as a social good.

We conclude that the financial risk arising from the use of innovative pharmaceutical cancer technologies fails to meet the aforementioned criteria, thus raising questions as to the sustainability of commercial insurance for cancer treatment and suggesting the need for the state to take greater responsibility for covering this financial risk in the future.

**Supplementary Information:**

The online version contains supplementary material available at 10.1186/s13584-020-00426-w.

## Introduction

“In what, undoubtedly, is one of the most difficult times in their lives, individuals with cancer should be focused on getting the best care possible, not worrying about financial strain on their families,” *ASCO Chief Executive Officer Clifford A. Hudis* https://www.ascopost.com/News/57848

Pharmaceutical technologies are developing at a dizzying pace. In the last decade, we have seen tremendous progress in the development of new classes of drugs that have greatly improved outcomes for patients with certain cancers. Immune checkpoint inhibitors, for example, have improved the prognosis for many patients with once rapidly fatal cancers [1]. Not only drugs, other cancer care technologies, such as gene and cell therapies are developing at a rapidly pace. In the oncology world, the rate of innovation is accelerating and the prices of new drugs and therapies are rising at a rapid rate. Furthermore, some of the new drugs and therapies increase life expectancy, which usually increases the duration of their usage as well [1, 2].

In many countries, including Israel, new drugs have in recent years become significantly more expensive than their predecessors. Over the years, the number of drugs in Israel with a price of over NIS 10,000 per package has grown significantly, as has the average price of drugs [3]. Lomnicky et al. [4] compared the trends in drug expenditure by the Maccabi Health Services over a 16-year period and found that while cancer drugs accounted for only 6.8% of its total drug expenditure in 1998, their proportion rose steadily over time to 30.2% in 2014, making them Maccabi’s largest single drug class expenditure. They explain this increase as being due to the increasing number of approvals for high-priced biological and targeted cancer therapies (such as monoclonal antibodies and modern tyrosine kinase inhibitors). They argue that this trend is likely to continue following the inclusion of costly checkpoint inhibitors in the Israeli National Health Insurance services basket.

In their comment on Lomnicky et al. [4], Goldstein et al. [5] discuss some of the problems and future challenges that will arise from the growing costs of cancer care and in particular cancer drugs. The researchers conclude that with the arrival of new therapies, the future of cancer care is promising, but that it will challenge the ability to pay for treatment.

In this perspective, we claim that the financial risk attached to the use of innovative pharmaceutical cancer technologies fails to meet at least three of the insurable risk criteria, thus raising the question of what commercial pharmaceutical insurance actually covers.

## The role of insurance

According to the economics of insurance theory, the welfare benefit of insurance is that it protects against the potential financial losses from a risky event/investment. Adam Smith writes as follows on insurance: “The trade of insurance gives great security to the fortunes of private people, and by dividing among a great many that loss which would ruin an individual, makes it fall light and easy upon the whole society” [6].

The fundamental purpose of health insurance is to reduce the financial risk associated with healthcare *spending*, where risk is interpreted as a measure of the variation of healthcare *costs* faced by an individual or household. The role of health insurance is to protect against a catastrophic financial loss due to an illness or a health situation that creates the need for healthcare treatments and pharmaceuticals that may be tremendously costly [7–9]. The primary argument in favor of health insurance is that, when it fulfils its purpose, it improves the healthcare system’s functioning and consumer welfare. Insurance mediates between consumers and healthcare providers in the health services market. In the event of serious illness, the insured faces both the uncertainty of the disease and the costs of its treatment. The insured turns to the insurer to provide financial assistance and to help finance the most appropriate treatment [10]. This is all the more so in the case of cancer and oncology patients [11, 12]. The insurer, on the other hand, will offer a policy that takes into account the probability of an event occurring and the cost of the claim.

## The multilayer health insurance structure

**Fig. 1:**
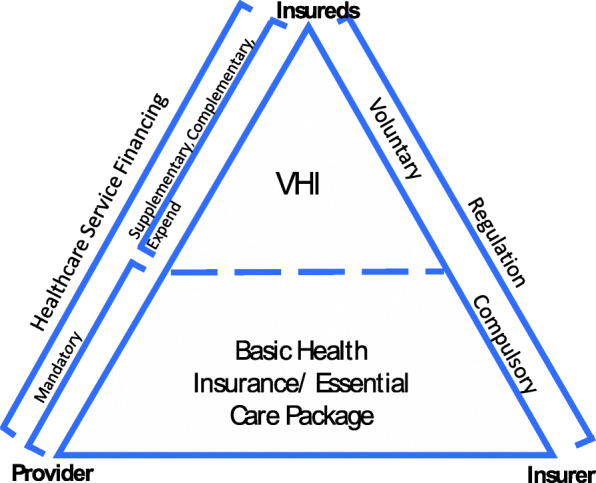
Representation of health care classification in multilayer health systems

A multilayer health insurance system is common in the OECD countries. The first layer of such structures is usually universal health coverage provided by means of various mandatory insurance schemes offered by regulated health plans or by private insurers. This layer provides basic health insurance, which usually covers essential health services, technologies and drugs. The Voluntary Health Insurance (VHI) layers are usually regulated separately from the compulsory basic insurance layer and their coverage often extends, supplements or complements the basic health insurance coverage (see Fig. 1) [13].

Even though the VHI market varies from country to country and the type of policies varies from insurer to insurer within the same country, the basic principles, which are derived from insurance theory, remain the same. VHI policies frequently play a supplementary role, by offering the same services included in the basic insurance scheme but with improved terms, such as providing faster access to care, greater choice of provider, improved amenities and reimbursement of co-payments and extending the services included in the basic insurance, such as additional physiotherapy or psychotherapy sessions. Another important role is to complement the basic insurance services by providing excluded benefits, such as innovative pharmaceutical cancer technologies. The importance of VHI lies in the welfare benefit it provides by increasing the scope of protection against the potential financial losses from a risky event/investment and reducing the financial risk associated with healthcare.

A comparison of Switzerland to the Netherlands is a good example of how multilayer health insurance systems can differ but still be based on the same principles. In both countries, there is basic compulsory health insurance that provides a relatively broad package of health services. The obligation of basic insurance is determined by law and is accompanied by the option for individuals to add additional layers of VHI. The Netherlands have instituted a multilayer health insurance scheme by means of a statutory health insurance system with basic coverage provided by universally mandated private insurance providers. The providers of the basic insurance are allowed to offer supplementary VHI coverage through a separate and well-regulated channel. The Netherlands has one of the highest levels of VHI coverage in the OECD countries with 84% of the total population opting for VHI. Switzerland has a decentralized (state-canton) health insurance scheme which includes mandatory health insurance provided by private insurers, who can also provide supplementary coverage. The Swiss VHI market share is around 29% of the population [13–15].

The Israeli health system is characterized by a three-layer health insurance scheme based on a national health insurance scheme managed by four health plans. In addition to the provision of the basic health package services, they are allowed to offer supplementary coverage; however, it cannot include lifesaving or life-prolonging treatments and therefore the health plans cannot satisfy the need we are discussing in this perspective. A third insurance layer is offered by commercial insurance companies and includes a variety of insurance options tailored to individual needs and a variety of different services, including pharmaceuticals. The VHI market share in Israel is relatively high, with 84% coverage among the population [16].

In order to complement the basic insurance coverage, VHI policies sometimes cover technologies that are excluded from the basic national healthcare packages. These policies usually insure against the risk of catastrophic financial loss due to the need for expensive innovative pharmaceutical technologies, which leads us to the concern regarding their insurability.

## The insurability of innovative pharmaceutical cancer technologies

In this section, we discuss concerns regarding the ability of VHI policies to cover the cost of innovative pharmaceutical cancer drugs and other cancer care technologies not included in the basic health package. The discussion leads us to conclude that an intervention by regulators will be necessary in countries with multilayer health insurance systems.

The question of whether losses are insurable by the VHI offered by commercial companies is one that can be answered. Since the work of Berliner [17], this question is often part of the analysis of insurance markets [18–21]. The concepts of insurability are uniformly applicable to any line of insurance and to both group and individual underwriting. He identified nine criteria, not all independent of one another, which define insurability. The criteria are categorized into three broad groups that classify risks in terms of actuarial, market, and societal conditions [17–21].

The actuarial criteria require that loss exposures be independent and that loss probabilities can be reliably estimated (randomness of loss occurrence); that *the maximum possible loss per event be manageable* in terms of insurer solvency^1^; that the average loss per event be moderate; that loss exposure be sufficiently large; and that the potential problems resulting from information asymmetry (i.e., moral hazard and adverse selection) not be excessive. The market criteria are satisfied if the *insurance premium* is adequate to provide cost recovery and is affordable for the target population and if the policy’s cover limits are acceptable. The societal criteria require that coverage be in accordance with *public policy and societal values* and with the legal restrictions governing coverage. (For a full list of the insurability criteria, see Additional file 1.)

In this perspective, we examine whether the coverage of innovative pharmaceutical cancer technologies is insurable according to three of the criteria (one from each of the three groups).

### Maximum possible loss per event must be manageable in terms of insurer solvency

The maximum possible loss is simply the maximum loss that can occur as the result of a risky event/investment. In the case of a building, and disregarding indirect losses, it is the replacement cost of that building. The situation is far more complex for innovative pharmaceutical cancer technologies whose cost is escalating and hard to predict.

In the US, it was found that the average launch price of a cancer drug, adjusted for inflation, increased by 10% annually—or an average of $8500 per year—from 1995 to 2013 [22]. It might be argued that this is the result of strong financial incentives for physicians and hospitals to use novel products, as well as the lack of therapeutic substitutes, which allows pharmaceutical manufacturers to set the prices of new products at or slightly above the prices of existing therapies, giving rise to an upward trend in launch prices [18]. Moreover, it is well known that the FDA approves technologies almost always based on safety and efficacy, with little consideration given to cost. Medicare must then, by law, provide any technologies that has FDA approval, thus weakening the ability to negotiate with pharmaceutical companies in order to lower costs. Furthermore, Lomnicky et al. [4] found that even when a health plan is widely implementing cost containment methods its expenditure on cancer care technologies rises significantly over time.

Goldstein et al. [5] point to future innovations that will increase the financial risk of becoming a cancer patient. One such area of innovation is targeted therapy and immunotherapy for precision oncology treatments. In targeted therapy, drugs directly attack the cancer by altering the expression of critical cancer genes identified using cancer genome profiling [23]. These targeted therapies hold great promise and it is expected that in the future they will be in common use in all types of cancer treatment. However, their cost is still unclear. Another example is related to biologic therapies. After patent expiry, we have traditionally seen major reductions in drug prices following the introduction of generic brands. This has been a fundamental assumption of healthcare payers in their budget calculations. However, many of the new cancer drugs are biologic agents, for which only biologically similar agents (and not generic brands) are possible and therefore the extent of competition and price reduction following patent expiration is uncertain [5].

In short, cancer care therapies in general, and cancer drugs in particular, have undergone dramatic change over the past decade. The complex biologic products being developed require a long and complex R&D process. These high development costs will pose a major challenge to payers, particularly when combination therapies are introduced. Together with the increase in public awareness of treatment options, this makes the *maximum possible loss per event* very hard to predict and to manage.

### Uncertainty in the level of insurance premiums

Insurability is becoming less feasible as the required premium increases. The market criteria require that the *insurance premium* be affordable to the target population, that it provide cost recovery and that the policy’s cover limits be acceptable [18–21]. The pure premium is equal to the expected value of the annual loss and—as a buffer against insurer ruin—a contingency loading to provide for adverse fluctuation in claim results. The greater the uncertainty, the higher will be the required contingency loading [20]. The need for innovative pharmaceutical cancer technologies involves at least three uncertainties: price, the period that the technology needs to be administered and the number of individuals that will need the technology. The first of these was discussed in the previous section.

The second relates to the fact that life expectancy of cancer patients is increasing over time. It has been found that patients who survive for 4 years following diagnosis are living longer, to the point that their life expectancy is approaching that of the general population. With increasing periods of survival since diagnosis, particularly for more lethal cancers, patients’ life expectancy tends to increase during the first three to 5 years immediately after diagnosis [24, 25]. In Israel, the National Cancer Registry reported that relative five-year survival^2^ of invasive cancer has increased significantly, based on a comparison between those diagnosed during 1996–2000 and those diagnosed during 2007–2011. This was the case for both Jews and Arabs and for both genders, with improvement in survival ranging from 13 to 20% [26]. The increase in life expectancy following diagnosis means that the length of time that expensive drugs are administered is also increasing. However, it is worth noting that this does not imply that a new pharmaceutical will by itself increase life expectancy; on the contrary, there is no clear-cut evidence regarding the contribution of these technologies to the increase in life expectancy [27], thus creating an additional source of uncertainty.

In parallel to the upward trend in cancer survival rates as a source of uncertainty, there is also variability in the incidence of invasive cancer, a third source of uncertainty. This is due to the fact that the observed cancer burden can be influenced by the diagnostic practice. Thus, new imaging techniques and other diagnostic methods can allow a cancer diagnosis to be made earlier in the disease course, they can detect nodal and metastatic involvement not recognized previously (thus, shifting the stage of cancer upstream), and they can even reveal some cancers that would otherwise not become evident clinically — a phenomenon now referred to as overdiagnosis. In the world of advanced medicine, there are several factors (such as the ability of the examinations to detect small irregularities and the threshold at which to label these as cancer) that can lead to rapid, iatrogenic swings in the reported incidence of cancer [28]. Moreover, although cancer rates remain high in high-income countries, they are plateauing or decreasing for the most common cancers, due to the decline in known risk factors and the improvement in screening and early detection, as well as improved treatment. In contrast, rates in many of the low- and middle-income countries are increasing due to the growing incidence of smoking, obesity, and physical inactivity [29, 30].

### Public policy and societal values

Meeting the public policy criterion requires that risk coverage be consistent with societal values. This means, for example, not insuring trivial risks and not providing any incentives for engaging in criminal acts [21].

The issue of whether the insurance coverage of innovative pharmaceutical cancer technologies accord with public policy is a complex one. On the one hand, health as a social good is a strong societal value in Israel, as it is in most countries, and therefore it is largely covered by public insurance through the basic healthcare package. In particular, public health insurance purports to protect against a catastrophic financial loss due to a serious illness such as cancer which creates the need for healthcare treatments and pharmaceuticals that may be tremendously costly. Therefore, society has created special mechanisms to publicly finance these costs [31]. Moreover, in Israel, as in in other countries such as Belgium, France and the Netherlands, the “solidarity” principle is paramount in health care financing. Equal access to health insurance is at the heart of the values held by the mutual insurers established in many countries [32]. On the other hand, since the new cancer treatments are liable to create a substantial financial burden on healthcare systems we are witnessing increasing pressure worldwide to prioritize anticancer technologies (by means of cost-utility analyses, value-based analyses or other methods) and to provide public coverage of only the high-priority ones, with the goal of limiting the burden on the public purse [33–35]. Therefore, in view of the expected financial burden, the principle of solidarity may be abandoned to some extent if society feels that these treatments should not be fully covered by public or private insurance.

There is a public debate in Israel over whether to adopt an automatic mechanism for updating the National Health Insurance (NHI) basket of services. At the center of this debate is the national expenditure on pharmaceuticals and medical equipment which has grown from 11.6% of healthcare expenditure in 1999 to 19.5% in 2018 (an increase of about NIS 10 billion). Moreover, the cost of adding a new oncology therapy to the NHI basket rose from an average NIS 34,000 per patient per year in 2008 to NIS 253,000 in 2019 [36]. Whether or not there will be an automatic update of the NHI basket, Israel is struggling to maintain public insurance coverage for its citizens. Examples of this include the risk-sharing mechanisms between the health plans and the pharmaceutical companies [36] and the health plans’ Exception Committees [37].

## Summary and conclusion

Following Lomnicky et al. [4] and Goldstein et al. [5], who raise the issue of growing expenditure on cancer treatment, including the cost of cancer drugs and other cancer care therapies, we claim that the financial risk arising from the use of innovative pharmaceutical cancer technologies fails to meet some of the insurable risk criteria, thus raising questions as to the sustainability of commercial insurance for this coverage.

There are three criteria for sustainability that may not be met. First, the uncertain trends in the cost of innovative pharmaceutical cancer technologies make the *maximum possible loss per event* very difficult to predict and to manage. Second, the uncertainty of the price, the period that a cancer care technology will be administered and the number of individuals that will need it make it difficult to predict future *insurance premiums.* Finally, pressure to limit *public coverage* will gradually increase as the possibilities of innovative pharmaceutical cancer technologies expand, thus transferring the burden onto commercial insurance, a phenomenon that is virtually impossible to predict accurately.

In conclusion, the difficulty in meeting the aforementioned criteria in the future represents a challenge to the insurability of innovative pharmaceutical cancer technologies. This will likely force commercial insurers to hedge their risk in order to avoid losses and to financially protect themselves from potential maximum losses, something that is already occurring. How they are doing so and what risk is actually covered by their policies will be examined in a forthcoming paper by the authors. Nonetheless, the situation in itself will require policymakers not only to deal with the rising prices of cancer care therapies, but also with the limiting of people’s private insurance alternatives. This will require out-of-the-box solutions, such as creating a mechanism similar to that employed in the case of private insurance of road accidents, which involved the creation of the Road Accident Fund (“Karnit” in Hebrew), a regulated fund whose role is to guarantee compensation for injury suffered in a car accident. The fund’s activity is financed by the compulsory car insurance premium, which is collected and transferred to the fund by the insurance companies. Another option could be a national medication and other technologies insurance law that will impose a progressive tax to finance the cost of advanced drugs and innovative technologies included in the National Health Insurance basket. Another possibility is a mechanism for updating the basket only with technologies that meet some threshold ratio of cost-effectiveness to be determined by the state. Regardless of the solution, it appears that the state will have no choice but to take greater responsibility in the future for the coverage of financial risk arising from the use of innovative pharmaceutical cancer technologies.

## Supplementary Information

**Additional file 1.** Insurability criteria and related requirements [13–17].

## Data Availability

Not applicable since no datasets were generated or analyzed during the current study.

## Footnotes

1 The key components in assessing the financial stability of an insurer are its solvency, capital adequacy and liquidity. *Solvency* is a measure of whether an insurer can cover its liabilities. It is important to note here that solvency issues are more likely to arise in relation to unexpected aspects of claims. In principle, the “expected” aspects of claims are manageable if they can be accounted for in the pricing of the policy’s premium. (Insurer Solvency Standard, https://pdfs.semanticscholar.org/283e/f13455b36f62ffedec3a8f680a07338f22a3.pdf)

2 The five-year relative survival index is the ratio of the percentage of cancer patients who have survived for 5 years since diagnosis to the percentage of the general population alive after 5 years (adjusted for gender, age and population group).
