# Supplementary material for: The insurability of innovative pharmaceutical cancer technologies
Source: Isr J Health Policy Res. 2020 Dec 21;9:69. doi: 10.1186/s13584-020-00426-w (PMC7751103; doi:10.1186/s13584-020-00426-w)
Supplement: Supplementary file 1 — Additional file 1. Insurability criteria and related requirements [13–17]. [file 13584_2020_426_MOESM1_ESM.docx]

**Additional file 1**

*Insurability criteria and related requirements* [13-17]

| Insurability criteria |  | Requirements |
| --- | --- | --- |
| Actuarial | 1. Randomness of loss occurrence 2. Maximum possible loss 3. Average loss per event 4. Loss exposure 5. Information asymmetry | 1. Independence and predictability of loss exposures 2. Manageable 3. Moderate 4. Loss exposure must be large 5. Moral hazard and adverse selection not excessive |
| Market | 1. Insurance premium 2. Cover limits | 1. Cost recovery and affordable 2. Acceptable |
| Societal | 1. Public policy 2. Legal restrictions | 1. Consistent with societal value 2. Allow the coverage |
